# El juego terapéutico como estrategia para reducir dolor y ansiedad en la atención pediátrica de urgencias

**DOI:** 10.23938/ASSN.1142

**Published:** 2025-10-03

**Authors:** Ana González-Díaz, Cristina Canals-Garzón, Juan Gómez-Salgado, Nora Suleiman-Martos, José Luis Gómez-Urquiza

**Affiliations:** 1 Facultad de Ciencias de la Salud Universidad de Granada Ceuta España; 2 Facultad de Ciencias de la Salud Universidad de Granada Granada España; 3 Departamento de Sociología, Trabajo Social y Salud Pública Facultad de Ciencias del Trabajo Universidad de Huelva Huelva España; 4 Programa de Posgrado en Seguridad y Salud Universidad Espíritu Santo Guayaquil Ecuador

## Sr. Editor:

El sistema sanitario español atraviesa actualmente un período crítico en relación a la saturación de los servicios de urgencias y emergencias, lo que exige soluciones innovadoras y eficientes en estas áreas. Por tanto, los profesionales deben afrontar retos relacionados con la gestión de recursos y la calidad asistencial^1^.

El ingreso en urgencias suele implicar procedimientos dolorosos, como la colocación de catéteres venosos periféricos, o la realización frecuente de exploraciones clínicas que forman parte habitual del abordaje diagnóstico y terapéutico. Sin embargo, la dinámica acelerada y la presión asistencial propias de las urgencias pueden intensificar las sensaciones de miedo, ansiedad y malestar en la población infantil^1^, vinculadas a factores personales, experiencias previas o características cultu-rales^2^.

La población infantil (denominada *niños* de aquí en adelante) es altamente sensible a este evento ya que, dependiendo de su edad, pueden no llegar a comprender realmente lo que está ocurriendo. La exposición repetida a estos procedimientos puede incrementar la carga de dolor y malestar, viéndose afectado tanto su bienestar emocional como su experiencia hospitalaria^3^.

Si el dolor no se controla de forma eficaz en los niños, puede desencadenar la activación del eje hipotálamo-hipofisario-suprarrenal, fobia a los pinchazos, problemas de sueño o incluso la evitación de procedimientos médicos^4^. Aliviar el dolor infantil es un aspecto fundamental en la atención pediátrica y constituye un derecho de estos pacientes; por ello, los hospitales tienen la responsabilidad de implementar estrategias para el control del dolor y la ansiedad^5^. Aunque existen métodos farmacológicos y no farmacológicos para el manejo del dolor en población infantil^6^, la mayoría no son utilizados por los profesionales sanitarios debido al tiempo, la dificultad de uso o el coste que supondrían^7^.

El juego es apropiado para la infancia y puede ayudarles en la adquisición del conocimiento sobre su enfermedad, además de lograr un afrontamiento más eficaz. El juego ayuda a captar y mantener la atención del niño, distrayéndolo del procedimiento doloroso o estresante. Involucrar al niño en actividades lúdicas no solo conlleva una distracción, sino que reduce la activación de las redes atencionales relacionadas con el dolor y la ansiedad^8^. Dado que los niveles de miedo y ansiedad en técnicas como la venopunción son elevados^2^, estamos desarrollando un estudio piloto que propone un juego breve basado en la teoría de redes atencionales^2^ de Posner, estructurado y adaptado a la edad del niño, para que redirija su atención y mejore su colaboración durante la venopunción. Esperamos obtener resultados prometedores que permitan dotar de relevancia a la terapia lúdica apoyada en un modelo científico^2^.

La incorporación de la tecnología como recurso terapéutico contribuye significativamente tanto a mejorar el comportamiento de los niños durante la realización de procedimientos invasivos como a reducir la sintomatología depresiva, por lo que sería muy beneficioso comenzar a utilizarla en las urgencias pediátricas. En ciertos grupos, el empleo de medios digitales ha demostrado tener un impacto terapéutico superior en comparación con los enfoques tradicionales^9^. Además, una de las ventajas del uso de dispositivos electrónicos radica en su elevado nivel de accesibilidad, especialmente en niños con limitaciones de movilidad, que facilitaría su participación en procesos recreativos^10^. Sin embargo, el verdadero valor del juego terapéutico radica en su sencillez y bajo coste: no requiere grandes infraestructuras ni personal adicional, sino únicamente la integración de actividades específicas dentro de la rutina asistencial, fundamentadas en una base psicológica y no solo lúdica.

En esta línea de investigación se han evaluado numerosas intervenciones para el manejo del dolor en procedimientos hospitalarios en niños, pero muchas no son adoptadas por su alto coste, demanda de tiempo o complejidad. El juego en urgencias pediátricas no debe verse solo como entretenimiento, sino también como una herramienta para reducir la ansiedad, el miedo y el dolor en la población atendida.

Desde el punto de vista de la teoría de redes atencionales, el juego es una estrategia que puede integrarse de manera relativamente sencilla y económica en la rutina asistencial sin necesidad de costosos equipamientos, ya que requiere recursos materiales mínimos. Su implementación no supone un gasto extra relevante: el material es básico, el tiempo de aplicación es breve y el personal sanitario puede asumirlo tras una formación mínima. Además, redirigir la atención del niño y, por tanto, conseguir generar menor ansiedad y miedo, disminuye la necesidad de intervenciones farmacológicas costosas.

Por lo mencionado anteriormente, sería beneficioso contar con programas especializados que capaciten al personal sanitario en la aplicación efectiva del juego como estrategia terapéutica, integrando técnicas de intervención, evaluación del impacto y adaptación en diferentes contextos clínicos. Lejos de implicar contrataciones adicionales o costes permanentes, esta capacitación puede realizarse dentro de la formación continua del propio personal, garantizando la sostenibilidad del modelo.

Asimismo, sería necesario disponer de recursos materiales que garanticen el correcto desarrollo del juego y una colaboración interdisciplinaria entre los diferentes profesionales del área sanitaria, además de contar con un espacio para poder desarrollar estos juegos al ingreso de los niños en el área de urgencias. No se requieren salas específicas ni nuevas infraestructuras, sino simplemente un espacio delimitado con mínima intimidad, lo que facilita su integración en cualquier unidad, incluso en hospitales con limitaciones estructurales.

En conclusión, el juego terapéutico en el ámbito de urgencias pediátricas no debe considerarse un recurso accesorio, sino una herramienta esencial para mejorar la calidad asistencial y la experiencia hospitalaria de la infancia y la adolescencia. Su eficacia para disminuir el dolor, la ansiedad y el miedo, junto con su bajo coste, facilidad de aplicación y fundamentación científica, lo convierten en una estrategia viable y sostenible. Apostar por su integración en la práctica clínica cotidiana supone no solo humanizar la atención sanitaria, sino también optimizar los recursos disponibles y garantizar un cuidado integral centrado en la niñez^3^.

## Data Availability

Se encuentran disponibles bajo petición al autor de correspondencia.
